# Concha Bullosa of the Inferior Turbinate: Report of Two Cases

**DOI:** 10.7759/cureus.15479

**Published:** 2021-06-06

**Authors:** Abdullah S Alkhaldi, Riyadh Alhedaithy, Yazeed Alghonaim

**Affiliations:** 1 College of Medicine, King Saud Bin Abdulaziz University for Health Sciences, Riyadh, SAU; 2 Otolaryngology Head & Neck Surgery Department, King Abdulaziz Medical City, Ministry of the National Guard – Health Affairs, Riyadh, SAU; 3 Otolaryngology Head & Neck Surgery Department, King Saud Bin Abdulaziz University for Health Sciences, Riyadh, SAU; 4 Otolaryngology, King Abdullah International Medical Research Center, Riyadh, SAU

**Keywords:** concha bullosa, inferior turbinate, inferior concha bullosa, rhinitis, rhinosinusitis

## Abstract

Concha bullosa (CB) is defined as pneumatization and the presence of air cells within the nasal turbinates. Inferior concha bullosa (ICB) is a rare anatomical variation of the lateral nasal wall, with only a handful of case reports published in the literature to date. In this article, we present two additional cases of ICB and a review of the literature regarding this rare anatomical variation.

## Introduction

Nasal conchae are important functional and anatomical structures of the lateral nasal wall. They are responsible for a variety of nasal functions, including filtration, humidification, lubrication, and the thermoregulation of inhaled air. Anatomically, superior and middle conchae are components of the ethmoid bone; however, the inferior concha is considered a separate bone. Pneumatization, or concha bullosa (CB), of the nasal turbinates is a normal anatomical variation of the lateral nasal wall. It is defined as the presence of air cells within the nasal turbinates. CB is most frequently reported in the middle turbinate and is considered rare in both the superior and inferior turbinates [1-2]. The etiology and mechanism of pneumatization of the nasal turbinates remain unknown. Inferior concha bullosa (ICB) was first recognized by Zinreich et al. in 1988 [3]. Although ICB is asymptomatic in most cases, symptoms can arise as a result of inferior turbinate hypertrophy or nasal cavity obstruction.

## Case presentation

Patient 1

**Figure 1:**
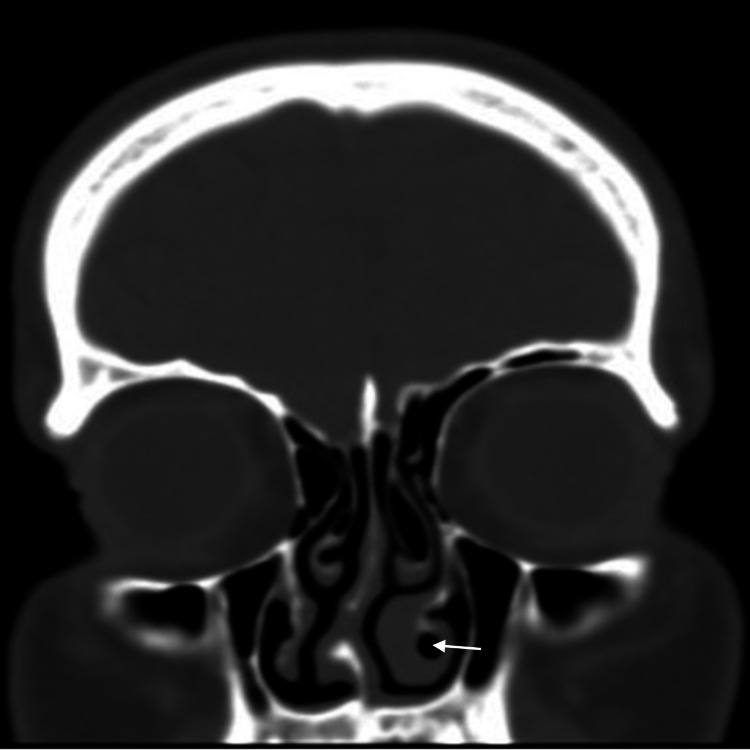
CT image of patient 1 (coronal view) Coronal view shows DNS to the right side, bilateral CB of the middle turbinates with right paradoxical middle turbinate, CB and hypertrophy of the left inferior turbinate (white arrow) DNS: Deviated Nasal Septum; CB: Concha Bullosa

**Figure 2:**
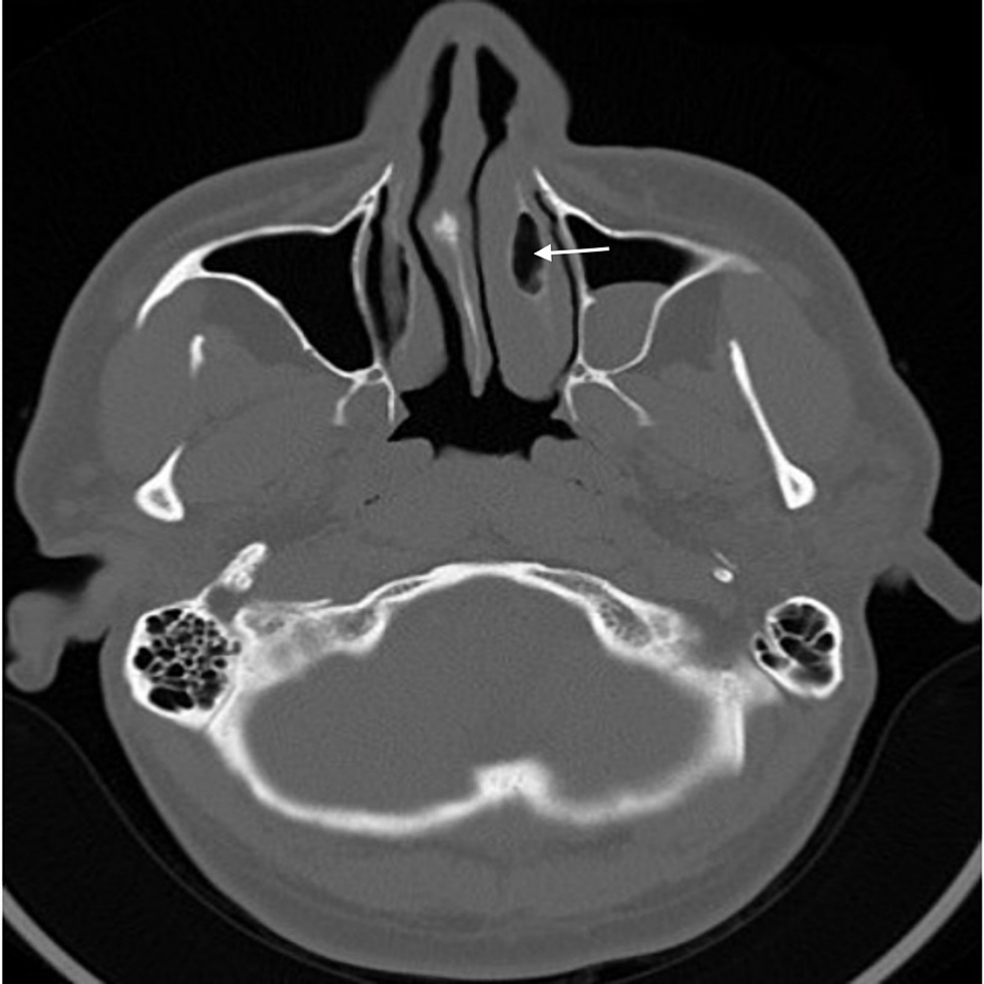
CT image of patient 1 (axial view) Axial view demonstrates CB of the left inferior turbinate (white arrow) CB: Concha Bullosa

A 32-year-old male presented to our otolaryngology clinic complaining of bilateral nasal obstruction and headache. He denied a history of allergies, trauma, or previous sinus surgery. Nasal endoscopy indicated a deviated nasal septum (DNS) to the right side and contralateral left inferior turbinate hypertrophy. For further evaluation, non-contrast computed tomography (CT) of the paranasal sinuses was performed. The CT showed DNS to the right, bilateral CB of the middle turbinates, paradoxical right middle turbinate, and CB of the left inferior turbinate (Figure 1 and Figure 2). The patient was initially treated medically with steroid and saline nasal sprays to improve the nasal mucosa hypertrophy. We followed the patient for a few months, and his symptoms had largely resolved. In case of symptom relapse, surgery will be offered to the patient.

Patient 2

**Figure 3:**
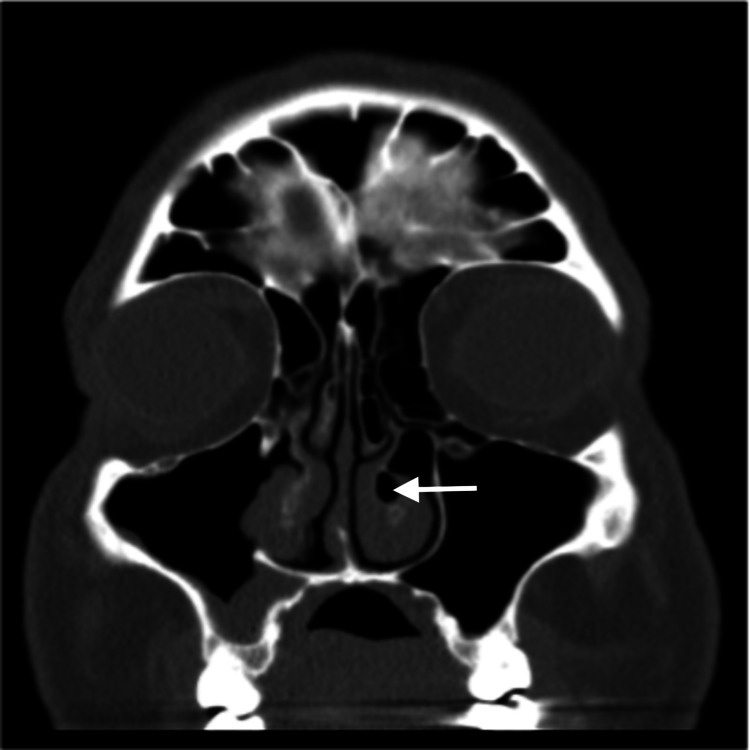
CT image of patient 2 (coronal view) Coronal view shows CB of both middle turbinates that is larger on the left side, CB of the left inferior turbinate (white arrow), and bilateral inferior turbinate hypertrophy CB: Concha Bullosa

**Figure 4:**
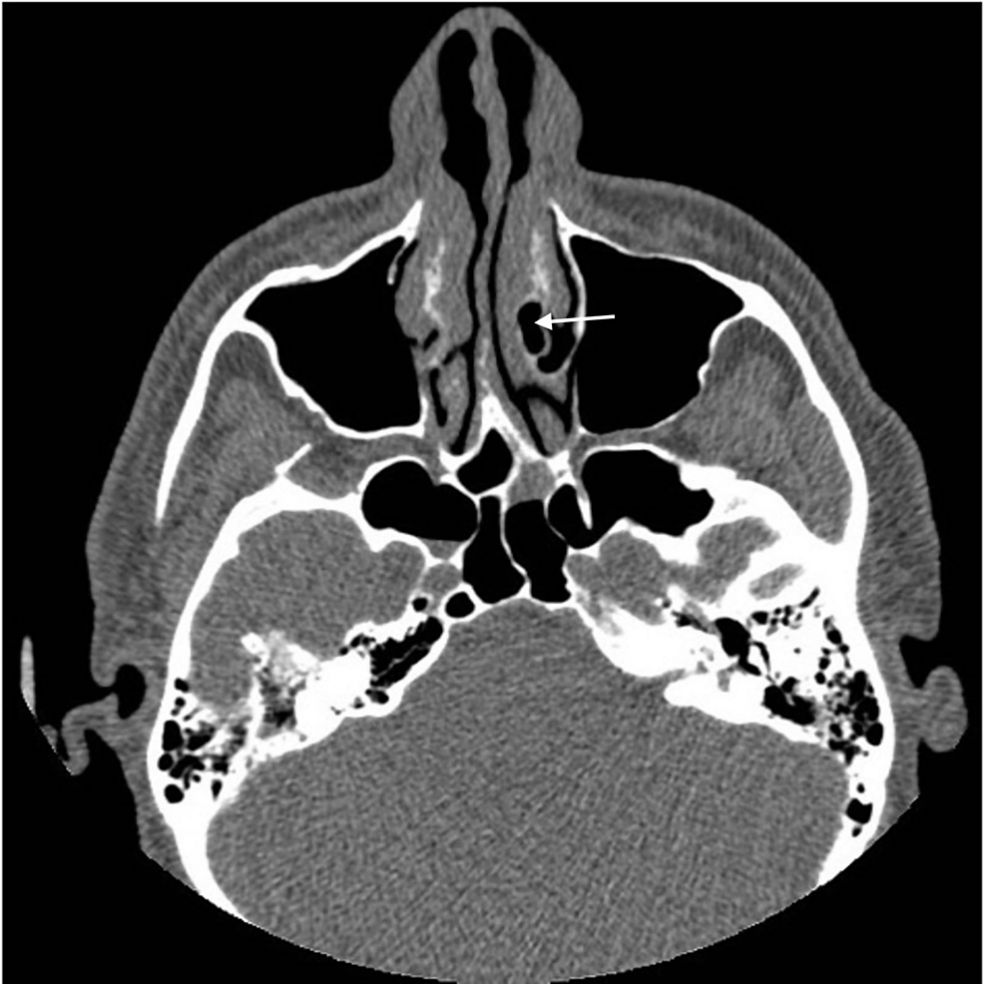
CT image of patient 2 (axial view) Axial view demonstrates CB of the left inferior turbinate (white arrow) CB: Concha Bullosa

A 28-year-old male presented to our otolaryngology clinic complaining of right nasal obstruction and headache. The patient has a history of recurrent rhinosinusitis and previously underwent a septoplasty. The nasal endoscopic examination showed bilateral inferior turbinate hypertrophy. For further evaluation, a non-contrast CT of the paranasal sinuses was performed. The CT indicated bilateral CB of the middle turbinate, predominantly on the left side, and CB of the left inferior turbinate with bilateral inferior turbinate hypertrophy (Figure 3 and Figure 4). The patient was medically treated with steroid and saline nasal sprays, with a good response.

## Discussion

The inferior turbinate is the largest of the turbinates and is mainly responsible for airflow direction, humidification, and the filtration of inhaled air. A swollen and enlarged inferior turbinate can obstruct nasal breathing. The enlargement of the inferior turbinate is rarely caused by ICB.

The mechanism of ICB formation is not fully explained in the literature; however, most of the broadly accepted theories regarding the etiology of ICB are related to its embryology. A few theories have been suggested to explain the formation of ICB. The first theory suggests that in fetal life, misinvagination of the epithelium occurs during ossification of the chondral framework of the inferior concha to a double lamella [4]. The second theory proposes that maxillary sinus disease results in air-filled cavities in the site of the inferior concha [5]. These two theories are proposed for the non-communicating type ICB. The third theory suggests that during fetal life, pneumatization of the maxillary sinus extends into the inferior concha, which can easily be seen on axial CT [1]. Unlike our two cases, a connection with the maxillary sinus is observed in CT for most ICB cases [6].

Patients with ICB can present with nasal obstruction, rhinorrhea, and headache. When the ICB is very large, it can compress nearby structures, affect the nasolacrimal duct, or obstruct the nasal cavity, resulting in headaches and rhinosinusitis. Both inferior turbinate hypertrophy and ICB can cause nasal obstruction, and it is essential to differentiate between them. A definitive diagnosis can only be achieved with CT of the paranasal sinuses. In general, ICB is often asymptomatic and usually diagnosed incidentally with CT imaging. In the literature, the incidence of ICB is 1% or less, with unilateral cases being more frequent [2,6-7].

Occasionally, treatment of ICB is not indicated unless the patient is symptomatic. Treatment goals include maximizing the nasal airway, preserving the function of the nasal mucosa, and minimizing complications. If the patient is symptomatic, medical treatment, such as vasoconstrictor drugs and intranasal steroid sprays, can be attempted first. The current two cases began with medical treatment to assess their response. However, medical treatment is occasionally ineffective in such patients, and surgery is required. Multiple surgical techniques have been described for the treatment of ICB, including the crushing of the ICB with forceps, out-fracture of the inferior turbinate, excision of the free edge of the inferior turbinate with angled scissors, sub-mucosal diathermy of the turbinate, and turbinoplasty with the use of the microdebrider [5,8-11]. In the case of a very large ICB, a lateral turbinectomy is indicated, as described by Dogru et al. [12]. Although his technique is easy and quick, it is contraindicated when there is a communication with the maxillary sinus because it may produce an inferior meatal antrostomy, which can cause mucociliary recirculation problems [13].

## Conclusions

ICB is a rare anatomic variation of the lateral nasal wall that is often asymptomatic and diagnosed incidentally with a CT scan. It may result in nasal obstruction, recurrent rhinosinusitis, and headaches. This study presented two cases of ICB that were treated medically without surgical intervention.
